# Folding to Curved Surfaces: A Generalized Design Method and Mechanics of Origami-based Cylindrical Structures

**DOI:** 10.1038/srep33312

**Published:** 2016-09-14

**Authors:** Fei Wang, Haoran Gong, Xi Chen, C. Q. Chen

**Affiliations:** 1 Department of Engineering Mechanics and Center for Nano and Micro Mechanics, AML, Tsinghua University, Beijing 100084, China; 2 Columbia Nanomechanics Research Center, Department of Earth and Environmental Engineering, Columbia University, New York, NY 10027, USA

## Abstract

Origami structures enrich the field of mechanical metamaterials with the ability to convert morphologically and systematically between two-dimensional (2D) thin sheets and three-dimensional (3D) spatial structures. In this study, an in-plane design method is proposed to approximate curved surfaces of interest with generalized Miura-ori units. Using this method, two combination types of crease lines are unified in one reprogrammable procedure, generating multiple types of cylindrical structures. Structural completeness conditions of the finite-thickness counterparts to the two types are also proposed. As an example of the design method, the kinematics and elastic properties of an origami-based circular cylindrical shell are analysed. The concept of Poisson’s ratio is extended to the cylindrical structures, demonstrating their auxetic property. An analytical model of rigid plates linked by elastic hinges, consistent with numerical simulations, is employed to describe the mechanical response of the structures. Under particular load patterns, the circular shells display novel mechanical behaviour such as snap-through and limiting folding positions. By analysing the geometry and mechanics of the origami structures, we extend the design space of mechanical metamaterials and provide a basis for their practical applications in science and engineering.

Origami, the art of folding a sheet into a 3D structure, has recently gained extensive attention in science and engineering^1^,^2^. Unique transformational abilities make origami structures widely applicable in fields such as self-folding machines^3^,^4^, aerospace engineering^5^,^6^, and biomechanics^7^,^8^. Although the fundamental relationships of a single origami unit (e.g., a unit of Miura-ori, or water bomb pattern) are understood, geometric relations when these units constitute “modular origami^9^” should also be understood. Among the many possible research directions in modular origami, the fundamental problem of designing 2D origami tessellations corresponding to desired 3D surfaces is still being studied. This “inverse” design issue has long aroused dissatisfaction^10^, but significant progress has been made recently^11^,^12^, whereas more design methods are still needed for various crease patterns^13^,^14^. The mechanics of origami is also of great interest^15^ and has substantially enriched the potential applications of mechanical metamaterials^1^,^16^. Novel stiffness and Poisson’s ratio possibilities, as well as bi/multi-stable properties, are studied for numerous origami patterns^2^,^17^,^18^ to facilitate their potential applications in mechanical actuators and energy absorption^19^,^20^,^21^. Much of the literature is concerned with origami mechanics of the “rectangular” or “cuboid” configuration^15^,^17^, and studies on relatively complicated configurations (such as shell structures) are limited. Under some circumstances, deformation modes that involve both folding of creases and bending of plates are considered^22^,^23^,^24^. In many cases, the thickness of the constituent plates cannot be ignored^6^,^25^,^26^. To maintain rigid foldability, thick plates are often separated and linked by thin films^6^ or extra hinges^27^. Recently, systematic kinematic models of thick origami are established^28^. There are stricter geometric compatibility conditions for thick plates (especially for those with periodic units) than zero-thickness plates.

In this paper, we propose a generalized in-plane design method that generates 2D Miura-ori tessellations according to the desired 3D cylindrical surfaces. Using the method, two fold types of Miura-ori crease lines can be unified in one reprogrammable procedure. The structural completeness conditions under which there are no gaps when plates are folded are developed. In particular, the mechanics of one type of cylindrical shell, namely, origami-based circular shells (OCSs), are investigated. First, the collision conditions and auxetic properties of the OCSs are explored. The unique mechanical responses to different loading patterns are demonstrated theoretically and simulated numerically. Moreover, by incorporating elastic properties into the plates, inhomogeneous deformation of the OCSs under radial line forces is numerically simulated.

## Results

### Generalized in-plane design method for cylindrical structures

**Figure 1:**
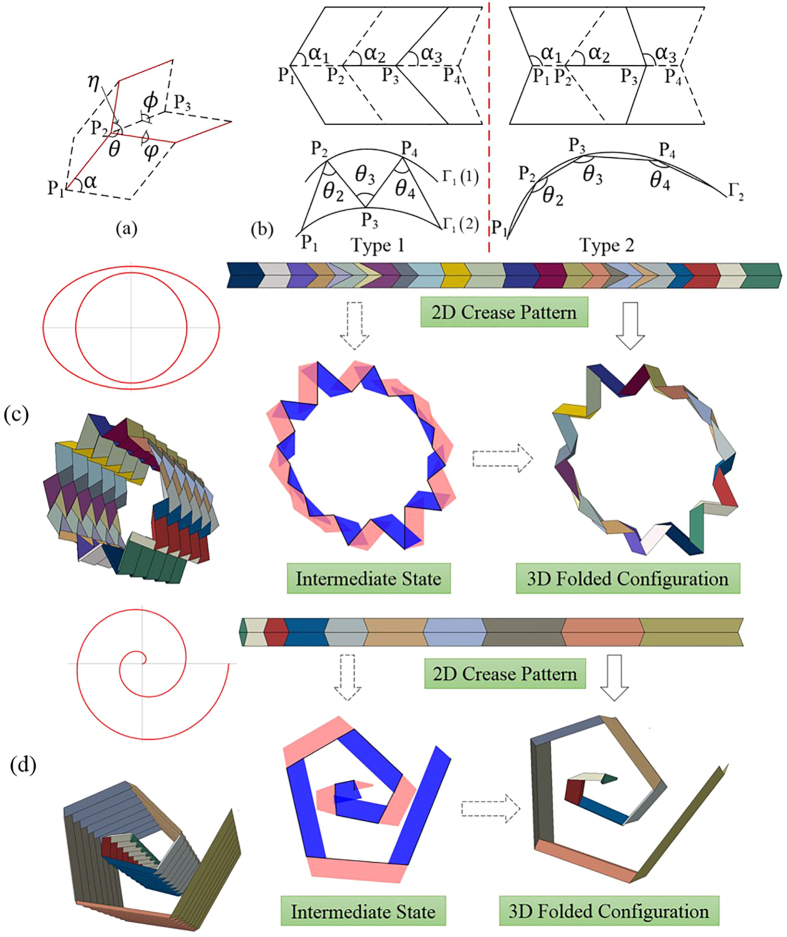
The in-plane design method using generalized Miura-ori units to form approximate cylindrical surfaces. (**a**) One unit of Miura-ori. Folds 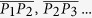 are “mainlines”. (**b**) Two types of crease patterns. Type-1: Vertices are picked one-by-one on two target curves Γ_1_(1) and Γ_1_(2). At every vertex, *α*_*i*_(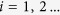) is an acute angle. In a folded configuration, *θ*_*i*–1_ and *θ*_*i*_ lie on different sides of fold 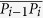. Type-2: Vertices are chosen on one target curve Γ_2_. *α*_*i*_(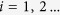) is acute and then obtuse. In a folded configuration, *θ*_*i*–1_ and *θ*_*i*_ lie on the same side of fold 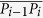. (**c**) An example of a Type-1 3D configuration. The target curves are a circle and an ellipse, respectively. (**d**) An example of a Type-2 3D configuration. The target curve is an Archimedes spiral.

Inverse origami design problems have been studied. 2D crease patterns and their corresponding quadrilaterals generated by previous methods^11^,^12^ are generally designed in the form shown in the left column of Fig. 1b. In this paper, another design method is proposed that can generate two types of crease patterns. A Miura-ori unit is shown in Fig. 1a. Fundamental geometric relations exist among the dihedral angles (*ϕ, φ*) and line angles (*θ, η*), in which *ϕ* (or its supplementary angle *ψ*) is chosen as the actuation angle during the folding/unfolding process in this study. The first step of the method is to choose in-plane vertices on/outside the directrix of the cylindrical surface, as shown in Fig. 1b. The folds connected by these vertices (*P*_1_, *P*_2_, *P*_3_ … in Fig. 1a,b) are called “mainlines” (see the black lines in the intermediate state of Fig. 1c,d). The 3D folded configuration that approximates the cylindrical surface of interest is the “prototypical configuration” relative to the 2D and other 3D configurations during folding. The prototypical angles, *θ*_*i*_^*P*^ (*i *= 1, 2, 3 …), combining the pre-defined height of the quadrilaterals *h* and the prototypical actuation angle *ϕ *^*p*^ constitute all of the independent parameters of the design method. The constant parameter *α* in Fig. 1a is then determined inversely. The values of *θ*_*i*_^*P*^ are located in-plane, and the orderly quadrilaterals are first formed in an “intermediate state” (this state does not exist in the actual folding process) and then “folded” to 3D space (Fig. 1c,d). More examples generated by the method are presented in Supplementary Information (SI).

Chen *et al*.^28^ developed a method to analyse the kinematics of thick origami. Their method is adopted here to investigate the structural completeness conditions of the two fold types discussed above. The conditions here refer to the folding case in which there are no gaps between thick plates. For infinitely thin Miura-ori, a spherical linkage is sufficient to model the kinematics, whereas for thick origami, other types of linkages (such as a spatial 4R-linkage) are necessary. Generally, the distances between the axes of creases are denoted by *α*_*i*_ (*i* = 1–4). According to the constraints of Bennett linkages^28^,


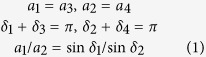


where *α*_*i*_ (*i* = 1–4) are the line angles divided by the crease lines (Fig. S2). For Miura-ori, the line angles satisfy *δ*_1_ = *δ*_2_. Therefore, the following relation is obtained:


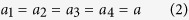


**Figure 2:**
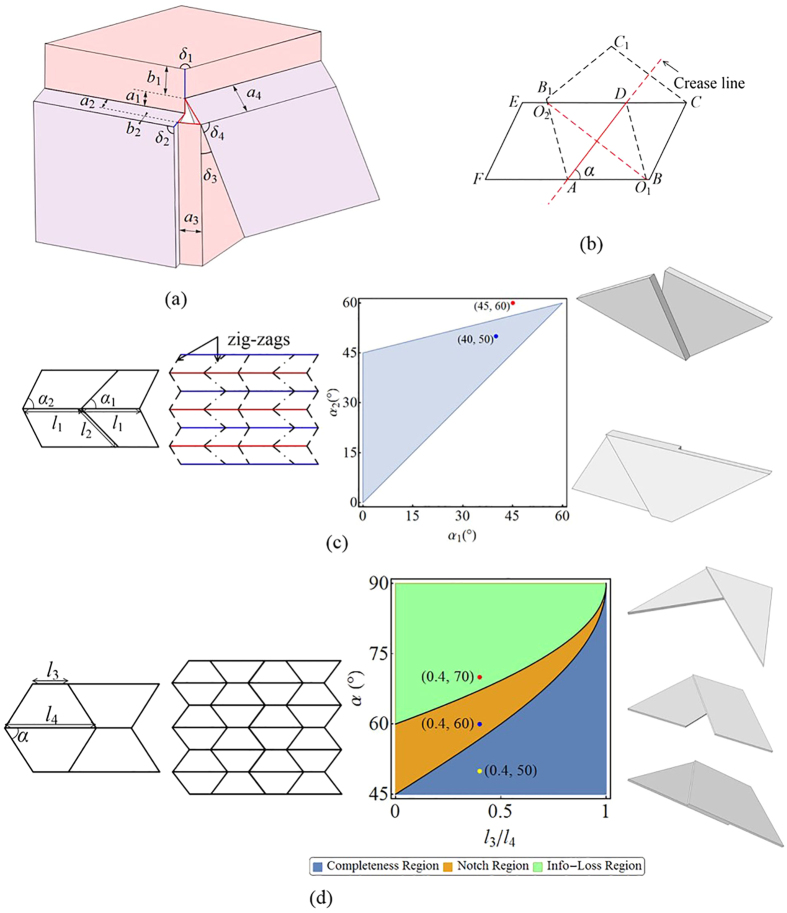
Generalized criteria of compatibility for Miura-ori structures with finite thickness. (**a**) A general thick origami structure with 4*R* Bennett linkage. Link lengths (red lines) *a*_*i*_(*i* = 1–4) are “effective” thicknesses of the 4 plates; the plates are connected with extra thicknesses (blue lines) *b*_1_ and *b*_2_. *δ*_*i*_(*i* = 1–4) are line angles. (**b**) Folding the Miura-ori structure along fold 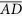, with 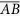 and 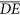 intersecting at *O* (that is, *O*_1_ on 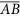 and *O*_2_ on 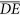). For thick Miura-ori structures, the overlapped parts Δ*ADO*_1_ and Δ*ADO*_2_ would be removed for a particular thickness in the third dimension. (**c**) An example of a Type-1 periodic thick Miura-ori structure. The red lines are “mainlines”. The *α*_1_ − *α*_2_ region plot provides the compatible value range for periodic thick Miura-ori structures when *l*_1_/*l*_2_ = 1. The red and blue dots correspond to the two thick cut-off plates on the right, respectively. (**d**) An example of a Type-2 periodic thick Miura-ori structure. All of the quadrilaterals are congruent isosceles trapezoids. The three dots in the respective regions correspond to the three thick cut-off plates. The right-top plate has lost the original geometric information *l*_4_ and cannot be used to constitute periodic thick origami.

As shown in Fig. 2a, extra thicknesses *b*_*i*_ (*i* = 1, 2) are necessary to connect the plate-crease-plate to ensure kinematic compatibility. For periodic Miura-ori, *b*_1_ = *b*_2_ = a should be satisfied because larger *b*_*i*_ hinders flat-foldability whereas smaller *b*_*i*_ leaves gaps in the structure when completely folded (*ϕ* = 0°). Note that plates are embedded into the neighbouring plates with thickness *α*_*i*_ when completely folded. Therefore, proper cutting of materials is necessary to ensure geometric compatibility. Specifically, as shown in Fig. 2b, to guarantee that no gaps exist after folding, side 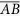 should intersect with side 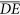, where the point of intersection between the two lines is *O*_1_ on 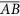 (or *O*_2_ on 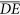). This condition requires the following inequality to be satisfied:


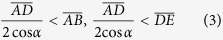


Using the above criterion, we present one specific example to discuss structural completeness conditions of the thick counterparts of these two fold types (Fig. 2c,d). The two patterns are generated naturally using the aforementioned method. In the first pattern, parameters *α*_*i*_ and *l*_*i*_ (*i* = 1, 2) should satisfy the following constraints to ensure the existence of the intersection point:


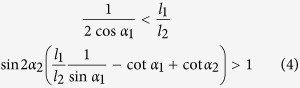


Specially, equation (4) reduces to linear constraints: 0 < *α*_1_ < 60° and 4*α*_2_ − *α*_1_ < 180° for *l*_1_ = *l*_2_ = *L*. A counterexample that loses completeness is shown in Fig. 2c. In the second pattern, the thick counterparts only maintain completeness when *α* < 45°, regardless of the ratio *l*_3_/*l*_4_. When *α* > 45°, constraint 1 − *l*_3_/*l*_4_ > 4 cos^2^*α* causes the remnant part to completely lose the original geometric information and no longer constitutes periodic thick origami; 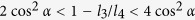 causes notches in the thick origami formed. Corresponding derivations are shown in SI.

### Geometry and mechanics of OCSs

**Figure 3:**
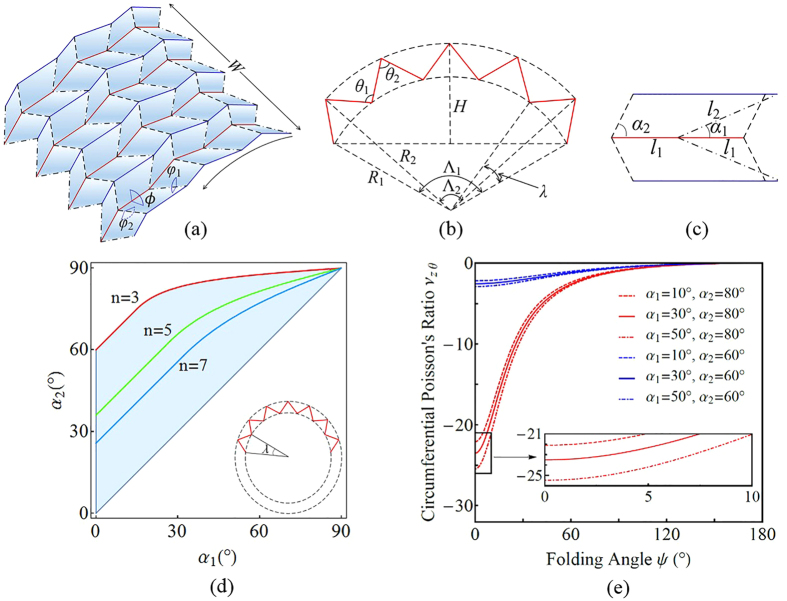
Geometric properties of the OCSs. (**a**) A 4 × 3 folded configuration generated by the method. (**b**) “Mainlines” and corresponding parameters. (**c**) An incompatible example when *α*_1_, *α*_2_ and *l*_1_/*l*_2_ are improperly collocated. (**d**) Collision conditions of OCSs in the circumferential directions. Given *n*, the condition is determined by *α*_1_ and *α*_2_ (*l*_1_/*l*_2_ = 1). The use of angle pairs (*α*_1_, *α*_2_) in the enclosed region guarantees that the OCSs do not collide. The small regions are subsets of larger regions. (**e**) Poisson’s ratio *ν*_*zθ*_ as a function of *ψ*.

Origami-based cylindrical shells are naturally generated using the developed in-plane method. Here, the geometry and mechanics of a specific case (i.e., OCSs) with the pattern shown in Figs 2c and 3 are investigated. With a pre-chosen constant parameter *h* and prototypical variables *ϕ*^*p*^, *θ*_*1*_^*p*^ and *θ*_*2*_^*p*^, all of the other constants (*l*_1_, *l*_2_, *α*_1_ and *α*_2_; *α*_1_ < *α*_2_) are determined. In 3D folded configurations, “mainlines” (the solid red lines in Figs 2c and 3a,b) are along the circumferential direction, and vertices on them are regularly distributed on two concentric circular surfaces with radii *R*_1_ and *R*_2_. The variables of OCSs with *m* × *n* unit cells are described in terms of the actuation angle *ψ* as:


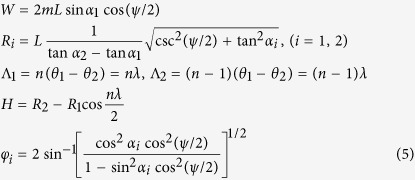


where *λ* is the central angle per unit cell in the circumferential direction and *θ*_*i*_ (*i* = 1, 2) are given by:


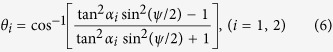


When the length ratio *l*_1_/*l*_2_ is given, to ensure geometric compatibility (Fig. 3c), the following constraints should be satisfied: 0 < *α*_1_ < *α*_2_ < 90° for *l*_1_/*l*_2_ ≥ 1; *α*_1_ and *α*_2_ lie within the region enclosed by 0 < *α*_1_ < *α*_2_ < 90° and 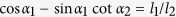 for *l*_1_/*l*_2_ < 1. In the following, the condition *l*_1_ = *l*_2_ = *L* is adopted to simplify analysis.

As an OCS is folded and the circumferential number *n* increases, it may not be intuitively clear what happens when the OCS reaches 360° in the circumferential direction and physical interference occurs. We study the collision conditions by characterizing the magnitude and monotonicity of the central angle *λ*. Theoretical results (see equations (18, 19) in Methods) reveal that when *α*_1_ + *α*_2_ < 90°, *λ* monotonically changes during folding. When *α*_1_ + *α*_2_ > 90°, however, *λ* varies non-monotonically and reaches the maximum value at 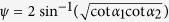, followed by a gradual decrease to 2(*α*_2_ − *α*_1_). Based on this monotonicity, the collision conditions of the OCSs are obtained. As shown in Fig. 3d, the angle pair (*α*_1_, *α*_2_) in the enclosed region guarantees that OCSs maintain foldability throughout the entire folding process. The boundary curves are linear (2*n*(*α*_2_ − *α*_1_) = 360°) when *α*_1_ + *α*_2_ < 90°, whereas they change to


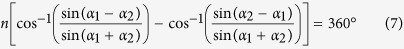


when *α*_1_ + *α*_2_ > 90° because of the non-monotonicity of *λ*.

The isometric deformation of OCSs can be quantified using the axial and circumferential strains, *ε*_*z*_ = *dW*/*W* and *ε*_*θ*_ = *dR*_1_/*R*_1_ + *d*Λ_1_/Λ_1_, respectively. The smaller radius, *R*_1_, is chosen to characterize the strain because the endpoints of *R*_1_ reach the outer edges of the structure in the circumferential direction (Fig. 3b). Poisson’s ratio is then extended to curved shell structures as *v*_*zθ*_ = −*ε*_*θ*_/*ε*_*z*_, which is obtained as follows:


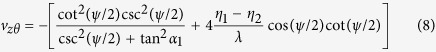


where


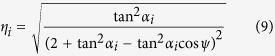


Figure 3e shows the variation of *v*_*zθ*_ as a function of *ψ* for selected set values of *α*_1_ = 10°, 30°, and 50°, and *α*_2_ = 60° and 80°. The OCSs are shown to be auxetic, with negative *ν*_*Zθ*_ monotonically increasing to 0 at *ψ* = 180°. Additionally, *ν*_*Zθ*_ has only a moderate dependence on *α*_1_ for a given *α*_2_; however, it is very sensitive to *α*_2_.

Then, we consider the mechanical responses of OCSs under radial force *F*_*r*_ and axial force *F*_*a*_, respectively. Models of rigid plates connected by linearly elastic torsional springs with particular initial folding angles are implemented. A constant *k*, which represents the torque required to twist one spring of unit length over one unit radian, is used to characterize the elastic properties of the springs^15^. The number of periodic units (*m* and *n*) should be considered because of the “boundary effect”. The strain energy *U* and external work *T* associated with an OCS are:


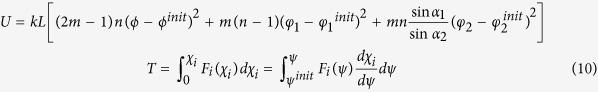


where the superscript “init” represents the initial states of corresponding quantities, whereas *F*_*i*_ and *χ*_*i*_ (*i* = *r, a*) are the radial and axial forces and their associated displacements, respectively. According to the principle of minimum potential energy, i.e., *δ*(*U* − *T*) = 0, corresponding balanced forces are then obtained (see SI).

**Figure 4:**
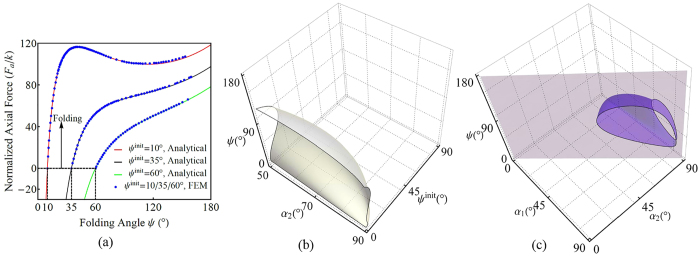
Snap-through transitions of a 9 × 5 rigid OCS under axial forces. (**a**) Analytical and FEM-predicted force-displacement curves for *α*_1_ = 45°, *α*_2_ = 60° and *ψ*^init^ = 10°, 35° and 60°. A small value of *ψ*^init^ induces snap-through transitions. (**b**) A zero-equipotential surface of instantaneous stiffness (∂*F*_*a*_/∂*ψ*) in *α*_2_ − *ψ*^init^ − *ψ* space, (*α*_1_ = 45°). (**c**) A zero-equipotential surface of instantaneous stiffness (∂*F*_*a*_/∂*ψ*) in *α*_1_ − *α*_2_ − *ψ* space (*ψ*^init^ = 18°). The surface does not exist in the lower triangular region (separated with the upper region by plane *α*_1_ = *α*_2_) because *α*_1_ < *α*_2_. In (**b**) and (**c**), any straight-line perpendicular to the bottom plane (the *α*_2_ − *ψ*^init^ plane in (**b**) or the *α*_1_ − *α*_2_ plane in (**c**)) represents a folding/unfolding process. Crossing of such a straight line and the equipotential surface indicates a snap-through transition or merely a load-softening phenomenon (when they are tangent).

Snap-through transitions of OCSs arise from the axial forces, which are induced by the existence of inflection points in the mechanical energy. Fig. 4a shows the *F*_*a*_ − *ψ* relationship for selected values of *α*_1_ = 45°, *α*_2_ = 60° and *ψ*^init^ = 10°, 35°, and 60°. Apparent snap-through appears for small *ψ*^init^. To further characterize the snap-through and hysteresis effects^17^ and alter these effects by redesigning the folds, we obtain 3D zero-equipotential surfaces of instantaneous stiffness (i.e., ∂*F*_*a*_/∂*ψ* = 0) by choosing and controlling the relevant variables. Fig. 4b,c show the surfaces in *α*_2_ − *ψ*^init^ − *ψ* and *α*_1_ − *α*_2_ − *ψ* space, respectively. Radial loads induce another important phenomenon: the existence of a limiting folding position *ψ*_*c*_ (*ψ*_*c*_ equals neither 0 nor 180°). When *ψ* → *ψ*_*c*_, *F*_*r*_(*ψ*_*c*_) → ∞. Notably, *ψ*_*c*_ is independent of *ψ*^init^, which indicates that even when OCSs are flat-foldable, we can adjust the foldability by choosing specific crease patterns, load methods, and boundary conditions. For both axial and radial loads, excellent agreement between the analytical and FEM predictions is obtained. More information can be found in SI.

**Figure 5:**
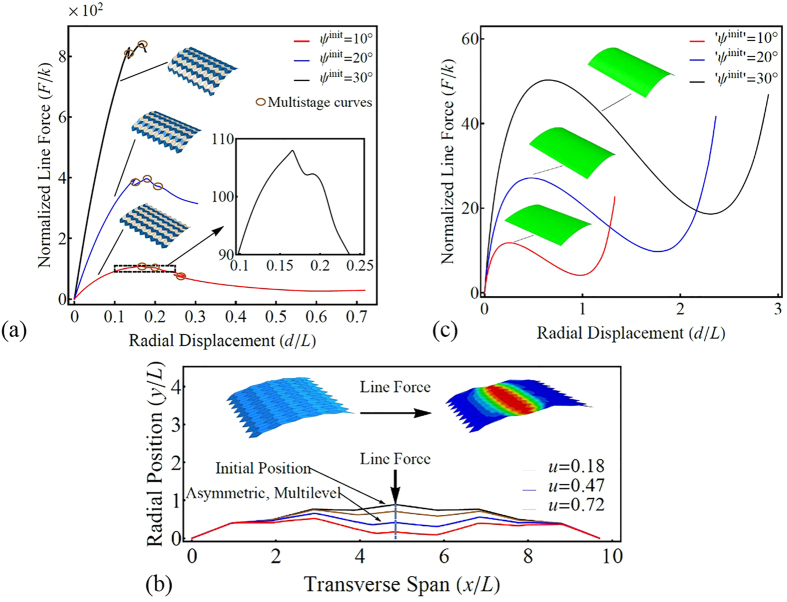
Mechanical responses of elastic OCSs. (**a**) Force-displacement curves of an elastic 9 × 5 OCS with *α*_1_ = 45° and *α*_2_ = 60° under a radial line force acting on the shell roof. The initial folding angles are *ψ*^init^ = 10°, 20° and 30°. (**b**) Inhomogeneous deformations of a “mainline” in the elastic OCS under different normalized displacements *u*, displaying multi-stage behaviours. (**c**) Force-displacement curves of the EHCSs.

The load-bearing capability and elastic stability of shell structures have received considerable attention. Several recent studies^29^,^30^,^31^ explored inhomogeneous deformation of origami-based structures or creased shells by considering the plates to be elastic instead of rigid. “Pop-through” or other types of defects in origami have been found to cause remarkable stiffness enhancement, and the elastic stability can be adjusted by modifying the crease patterns. Here, FEM is used to study the deformation of elastic OCSs subject to radial loads. The boundary conditions for the periphery along the axial direction are clamped. Simulated force versus displacement curves for three OCSs with different *ψ*^init^ = 10°, 20°, and 30° are shown in Fig. 5. The creases divide the OCS into multiple plates, and snap-through is initiated at the early stage of deformation (indicated by the circles in Fig. 5a). Multi-stage deformations in a cross section that characterizes the snap-through process are shown in Fig. 5b. For comparison, the force-displacement responses of the equivalent homogeneous cylindrical shells (EHCSs) (i.e., having the same volume of mass, radius, and central angle) are shown in Fig. 5c, revealing deformation different from OCSs. First, the OCSs are much stronger than their equivalent counterparts. Second, the load-displacement curves of the homogeneous shapes are much smoother, and the global load softening occurs much later.

## Conclusion and Discussion

This paper proposes a generalized design method for deployable cylindrical structures. By unifying two crease patterns in one reprogrammable procedure and studying the influence of thickness, the method provides a method to construct compact engineering structures. Additionally, the geometry and mechanics of Miura-ori based circular shells are investigated, providing further understanding of mechanical metamaterials, including auxetic properties, snap-through transitions, and limiting folding positions. Such unique and interesting properties of origami structures make them attractive for various applications in science and engineering.

Although the developed in-plane design method has been demonstrated to construct various origami-based cylindrical structures, it is desirable to determine whether the method can be extended to more complex structures such as undevelopable surfaces, even at a less approximate level. Furthermore, all the constituent units are Miura-ori, whereas some other crease patterns, such as water bomb^19^ and Resch patterns^32^, can also be adopted to form curved surfaces. Finally, further study on the mechanics of other types of origami shells is planned, especially in the areas of snap-through transitions and the stability of elastic origami.

## Methods

### Procedures of the in-plane method

**Figure 6:**
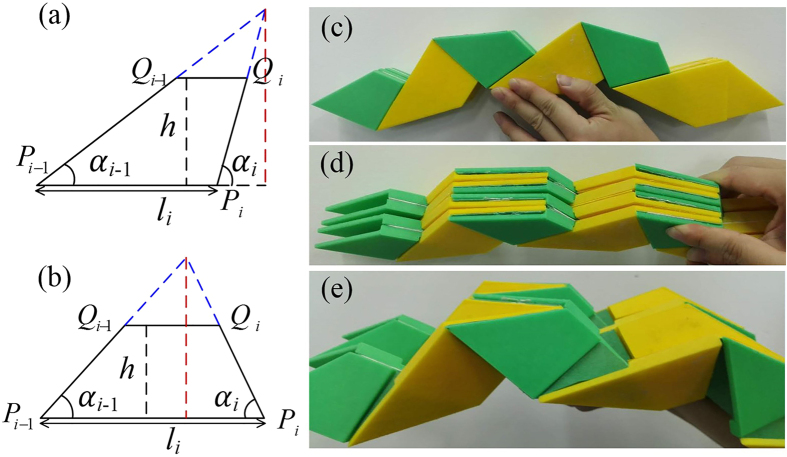
(**a**,**b**) Two types of trapezoids discussed in the model. The dashed red lines represent the admissible maximum height when the pre-chosen parameters are determined. (**c**,**d**,**e**) Experimental sample of the finite-thickness periodic OCSs displaying a cylindrical shape: (**c**) front view of the completely folded sample, (**d**) oblique view of the completely folded sample, (**e**) front view of the partly folded sample.

The method is called an “in-plane method” because all of the quadrilateral information is determined in-plane (i.e., the “intermediate state” in Fig. 1c,d). Open target curves (e.g., spiral curves) are used as examples to demonstrate the method; the method for closed curves (e.g., the circle and ellipse shown in Fig. 1c) can be obtained with some modification. The unified procedure of the two types of fold patterns is presented as follows:

1. Vertices *P*_*i*_ are chosen on two target curves (or one target curve) if a Type-1 (or Type-2) pattern is desired; thus, the values of 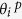 are determined naturally by the positions of *P*_*i*_. Using pre-chosen values of *φ*^*p*^ and *h*, the desired prototypical 3D configuration is obtained. The Miura-ori shape exhibits a single DOF; thus, according to the fundamental relations between *α, ϕ, φ, θ* and *η* of a Miura-ori shape, the values of *α*_*i*_ can be calculated as follows:
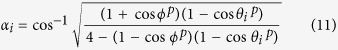
2. For the vertices chosen, the following parameters are determined: length of every “mainline” (i.e., 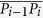) *l*_*i*_, and angle between every “mainline” and positive *x*-axis *ς*_*i*_ = *ς*_*i*−1_ + 180° − *θ*_*i*_. The primary function of the method is to locate the in-plane positions of every trapezoid *P*_*i*−1_*P*_*i*_*Q*_*i*_*Q*_*i*−1_ (see Fig. 6a,b and “Intermediate state” in Fig. 1), i.e., positions of point *Q*_*i*−1_ and *Q*_*i*_. Assuming that the vertices are chosen in a counter clockwise manner:

For the Type-1 pattern, when *P*_*i*−1_ is located on Γ_1_(1) and *P*_*i*_ on Γ_1_(2), the Cartesian coordinates of *Q*_*i*−1_ and *Q*_*i*_ are as follows:


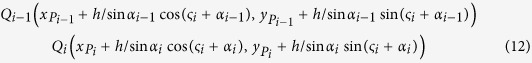


The next *P*_*i*−1_ is located on Γ_1_(2), whereas *P*_*i*_ is located on Γ_1_(1) (Fig. 1b,c); under this circumstance, the Cartesian coordinates of *Q*_*i*−1_ and *Q*_*i*_ are as follows:


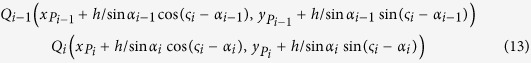


Repeating the above procedure generates all of the Type-1 trapezoidal information.

For the Type-2 pattern, when 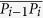 is the shorter bottom of the trapezoid’s two bottoms, the Cartesian coordinates of *Q*_*i*−1_ and *Q*_*i*_ are as follows:


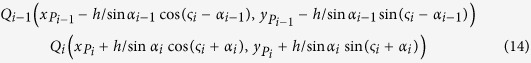


The next 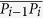 is the longer bottom of the trapezoid’s two bottoms (Fig. 1b,d), and the Cartesian coordinates of *Q*_*i*−1_ and *Q*_*i*_ are as follows:


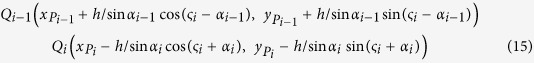


Repeating the above procedure generates all of the Type-2 trapezoidal information.

3.Because all of the trapezoids have been determined at the intermediate state, rotating them around their respective “mainlines”, for *ϕ*^*p*^/2 and −*ϕ*^*p*^/2, to the 3D configuration will achieve the desired configuration (see Fig. 1c,d). After repeating these symmetric units in the third dimension, the desired Miura-ori based cylindrical structures are obtained.

The parameter *h* is one of the pre-chosen parameters in the method. An excessively large value of *h* causes the two hypotenuses of the trapezoid to intersect (Fig. 6a,b). We specify a general criterion of choosing *h* to maintain compatibility. For the Type-1 crease pattern, the criterion is as follows:


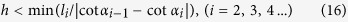


For the Type-2 crease pattern, the criterion is as follows:


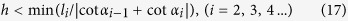


### Experimental models of periodic thick OCSs

Using the modelling method established by Chen *et al*.^28^, we present an experimental example of periodic thick OCSs. As shown in Fig. 6c,e, the 3 × 2 thick OCS has a cylindrical shape in the folded state. Following equation (2) and taking *b*_1_ = *b*_2_ = *a*, we demonstrate that no gaps remain after complete folding (Fig. 6d). The parameters of the sample are: *L* = *l*_1_ = *l*_2_ = 10 cm, *α*_1_ = 40°, *α*_2_ = 50°, and *a* = *b*_1_ = *b*_2_ = 0.5 cm.

### Collision conditions of OCSs

Monotonicity of the unit central angle *λ* is assumed to characterize the circumferential expanding ability of the OCSs:


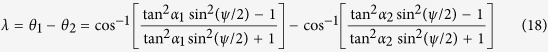


Thus,


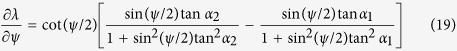


Monotonicity of the following function is required to estimate the variation of *λ*:


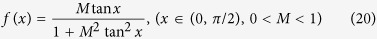


Further results indicate that under the assumption that 0 < *α*_1_ < *α*_2_ < *π/*2, *λ* increases (i.e., 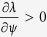) monotonically when *α*_1_* *+ *α*_2_ < *π*/2, whereas for *α*_1_ + *α*_2_ > *π/*_2_, there is only one 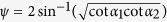 that results in 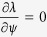, which causes *λ* to first increase to the maximum value and then decrease; when *ψ* → *π*, 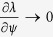, the OCSs fold completely, and *λ* approaches a constant value 2(*α*_2_ − *α*_1_). Thus, we obtain


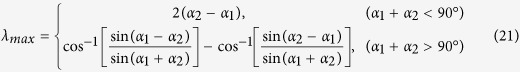


The OCSs do not self-overlap if and only if 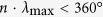, which are the collision conditions of OCSs (Fig. 3d).

## Additional Information

**How to cite this article**: Wang, F. *et al*. Folding to Curved Surfaces: A Generalized Design Method and Mechanics of Origami-based Cylindrical Structures. *Sci. Rep.* **6**, 33312; doi: 10.1038/srep33312 (2016).

## Supplementary Material

Supplementary Information
