# Supplementary material for: Folding to Curved Surfaces: A Generalized Design Method and Mechanics of Origami-based Cylindrical Structures
Source: Sci Rep. 2016 Sep 14;6:33312. doi: 10.1038/srep33312 (PMC5022034; doi:10.1038/srep33312)
Supplement: Supplementary Information [file srep33312-s1.pdf]

Supplementary Information for

Folding to Curved Surfaces: A Generalized Design Method and Mechanics of Origami-based Cylindrical Structures

Fei Wang [1], Haoran Gong [1], Xi Chen [2], Changqing Chen [1,*]

[1] Department of Engineering Mechanics, Centre for Nano/Micro Mechanics, AML

Tsinghua University, Beijing 100084, China

[2] Columbia Nanomechanics Research Centre, Department of Earth and Environmental Engineering, Columbia University

New York, NY 10027, USA

[*] Corresponding author. Tel/Fax: +86 10 62783488; Email: chencq@tsinghua.edu.cn (C.Q. Chen)

Cylindrical surfaces generated using the Method

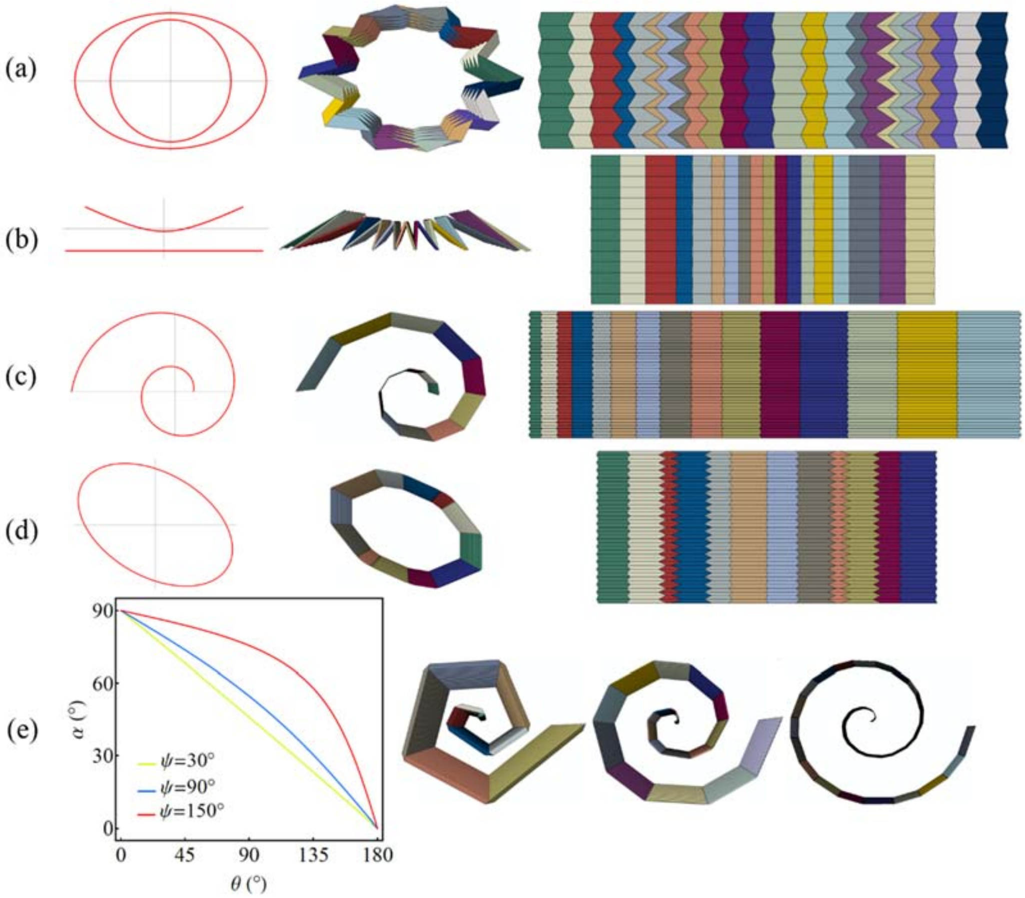

Figure S1 Some cylindrical surfaces by the in-plane method. (a) Type-1: target plane curves (a circle and ellipse), 3D origami configuration and 2D crease pattern. (b) Type-1: target plane curves (a hyperbolic curve and straight line), 3D origami configuration and 2D crease pattern. (c) Type-2: target plane curve (an equiangular spiral), 3D origami configuration and 2D crease pattern. (d) Type-2: target plane curve (an ellipse), 3D origami configuration and 2D crease pattern. (e) In $720°$ (4π radians), more points picked make the approximation more accurate. While this also leads larger θ_i^p prechosen. According to the fundamental relations of equation (11), smaller α_i are then obtained, leading the trapezoids formed "flatter".

Criterions of geometric compatibility for thick Miura-ori

The necessary and sufficient conditions for line segment $\overline{AB}$ to intersect with $\overline{DE}$, as can be seen in Fig. 2b, are:

$$\frac{\overline{AD}}{2\cos\alpha} < \overline{AB}, \frac{\overline{AD}}{2\cos\alpha} < \overline{DE} \tag{S1}$$

For an Type-1 Miura-ori shown in Fig. 2c, with constant parameters α_1, α_2, l_1, and l_2, equation (S1) has the form of:

$$\begin{aligned} &\frac{l_2}{2\cos\alpha_1} < l_1, \frac{l_2}{2\cos\alpha_1} < l^{''} \\ &\frac{l_3}{2\cos\alpha_2} < l_1, \frac{l^{'}}{2\cos\alpha_2} < l^{'''} \end{aligned} \tag{S2}$$

with $l^{'} = l_2 \sin\alpha_1 / \sin\alpha_2$, $l^{''} = l_1 + l_2 \cos\alpha_1 - l^{'}\cos\alpha_2$ and $l^{'''} = l_1 - l_2\cos\alpha_1 + l^{'}\cos\alpha_2$ being side lengths of the quadrilaterals (see Fig.S2a). Upon further simplification, we obtain the constraints for α_1, α_2 and l_1/l_2, as shown in equation (4). For an Type-2 Miura-ori shown in Fig. 2d, All the quadrilaterals are congruent. As shown in Fig. S2b, because of symmetry of the isosceles trapezoids, there are 3 cases after folding and cutting off materials. The first case is $\overline{CO_2} > \overline{O_2D}$, in which the finite-thickness plate maintain geometric completeness. This requires that $1 - l_3/l_4 < 2\cos^2\alpha$. The second case is $\overline{CO_2} > \overline{O_2D}$ in which there will be a notch left. This requires $2\cos^2\alpha < 1 - l_3/l_4 < 4\cos^2\alpha$ according to equation (S1). While the last case is that point O_2 locates on extension of $\overline{DC}$, which means $\overline{BC}$ will be cut off and geometric information will be lost(length of the bottom edge l_4 and height of the trapezoid h). Note that all the above circumstances are discussed when $\alpha > 45°$. Corresponding examples are shown in Fig. 2d.

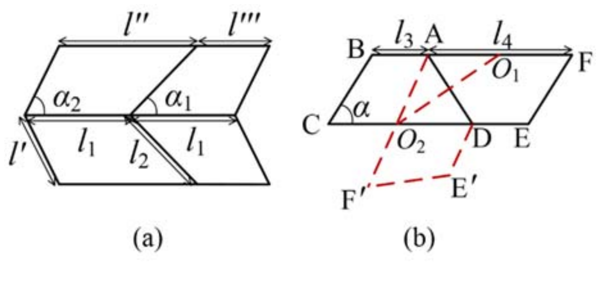

Figure S2 2 types of fold patterns when folding thick Miura-ori. (a) Type-1 Miura-ori, in which α_1, α_2, l_1 and l_2 are independent parameters. (b) Type-2 Miura-ori. Note that quadrilaterals $ABCD$ and

$ADEF$ are congruent isosceles trapezoids. The position of intersection point O_2 (or O_1 on $\overline{AF}$) after folding determines completeness of this type of thick Miura-oir.

Geometric relationships, compatibility and Poisson's ratio of OCSs

The 4-crease origami pattern studied in this paper has single DOF. With zero-thickness models, four fold lines of the OCSs meet at one vertex, acting as revolute joints to make the origami act as spherical linkages (Fig. S3). According to spherical trigonometry[1], Geometric relationships between the line angles and dihedral angles can be described as:

$$\begin{gathered} f_1 = \varsigma_1 + \varsigma_2 \\ f_3 = \varsigma_3 + \varsigma_4 \\ \cos f_2 = \left(\cos\Theta - \cos\delta_2 \cos\delta_3\right) / \left(\sin\delta_2 \sin\delta_3\right) \\ \cos f_4 = \left(\cos\Theta - \cos\delta_1 \cos\delta_4\right) / \left(\sin\delta_1 \sin\delta_4\right) \end{gathered} \tag{S3}$$

where $f_i\left(i=1\text{-}4\right)$ are dihedral angles formed plates of folded-state origami, $\delta_i\left(i=1\text{-}4\right)$ are corresponding line angles, $\varsigma_i\left(i=1\text{-}4\right)$ are auxiliary dihedral angles between surface *AOC* and four plates *DOA*, *AOB*, *BOC* and *COD*, respectively, and Θ is the line angle $\angle AOC$. The four auxiliary angles can be written as:

$$\begin{gathered} \cos\varsigma_1 = \left(\cos\delta_4 - \cos\Theta\cos\delta_1\right) / \left(\sin\Theta\sin\delta_1\right) \\ \cos\varsigma_2 = \left(\cos\delta_3 - \cos\Theta\cos\delta_2\right) / \left(\sin\Theta\sin\delta_2\right) \\ \cos\varsigma_3 = \left(\cos\delta_2 - \cos\Theta\cos\delta_3\right) / \left(\sin\Theta\sin\delta_3\right) \\ \cos\varsigma_4 = \left(\cos\delta_1 - \cos\Theta\cos\delta_4\right) / \left(\sin\Theta\sin\delta_4\right) \end{gathered} \tag{S4}$$

For Miura-ori pattern, line angles δ_i are either equal or supplementary to each other, that is:

$$\delta_1 = \delta_2 = \pi - \delta_3 = \pi - \delta_4 = \alpha_i \left(i=1,2\right) \tag{S5}$$

Where α_1 and α_2 are the 2 constant parameters of OCSs. By substituting equations (S4-S5) into S3, dihedral angles have the relations:

$$f_1 = 2\pi - f_3,\ f_2 = f_4 \tag{S6}$$

Note that dihedral angles are defined in the constraint $0 < f < \pi$, so we have

$$
\begin{gathered}
f_1 = f_3 = \phi \\
f_2 = f_4 = \varphi \\
\cos^2 \frac{\phi}{2} = \frac{1}{\tan^2 \alpha} \frac{1+\cos\Theta}{1-\cos\Theta}
\end{gathered}
\tag{S7}
$$

Because of α_1 and α_2, Θ and φ change alternately along the circumferential direction, while the dihedral angles ϕ keeps constant, as can be seen in Fig. 3a and 3b. So, the supplementary angle $\psi = \pi - \phi$ is adopted to characterize the folding process, taking into account that ψ increases as OCSs fold. Substituting Θ with $\theta_i\left(i=1,2\right)$, and ϕ with $\varphi_i\left(i=1,2\right)$ respectively, the central angle and radii have the relationships:

$$
\begin{gathered}
\theta_2 + 2\cdot\frac{2\pi-\theta_1}{2} + \lambda = 2\pi \\
R_1 \sin\frac{\lambda}{2} = l_1 \sin\frac{\theta_2}{2} \\
R_2 \sin\frac{\lambda}{2} = l_1 \sin\frac{\theta_1}{2}
\end{gathered}
\tag{S8}
$$

Expressing all the variables in terms of ψ, equations (5-6) are obtained.

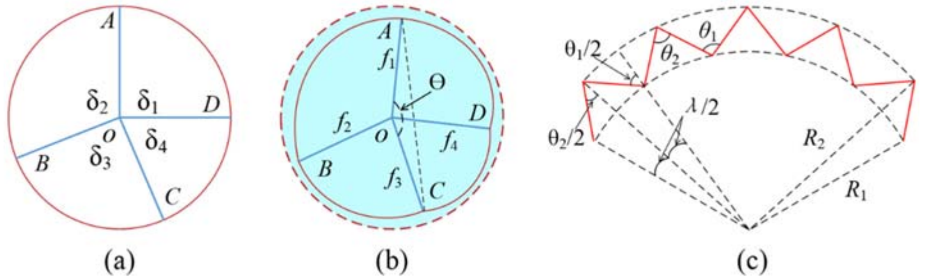

Figure S3 Geometric relations between folding parameters. (a) and (b) Crease pattern of a spherical linkage and corresponding its folded state. Relations between $\delta_i\left(i=1\text{-}4\right)$ and $f_i\left(i=1\text{-}4\right)$ are expressed in equations S3 and S4. (c) $\theta_i\left(i=1\text{-}2\right)$, unit central angle λ and radii $R_i\left(i=1,2\right)$ of an OCS.

To maintain geometric compatibility, the shortest length of all the quadrilateral sides in OCSs should be positive (see Figs. 2c and 3c), which requrires:

$$
l_1 + \left(l_2 \frac{\sin\alpha_1}{\sin\alpha_2}\right)\cos\alpha_2 - l_2\cos\alpha_1 > 0 \tag{S9}
$$

Together with the constraint $0<\alpha_1<\alpha_2<\pi/2$, Fig. S4 are obtained according to different ratios l_1/l_2.

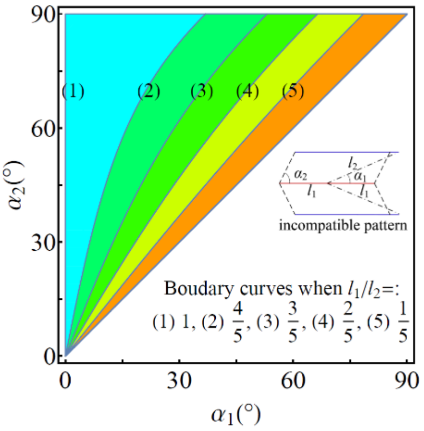

Figure S4 Geometric compatibility conditions of OCSs. Admissible regions of α_1 and α_2 associated with different values of l_1/l_2. Note that the small regions are subset of larger ones. The inset shows an incompatible example when α_1, α_2 and l_1/l_2 are improperly collocated.

In OCSs, the coupling of isometric deformation in axial and circumferential directions can be described by the variable $v_{z\theta}=-\varepsilon_\theta/\varepsilon_z$, which is an analogy of physical quantity Poisson's Ratio. The OCS's strain in the 2 directions are defined as follows:

$$\varepsilon_z=\frac{dW}{W}$$
$$\varepsilon_\theta=\frac{(R_1+dR_1)(\Lambda_1+d\Lambda_1)-R_1\Lambda_1}{R_1\Lambda_1}=\frac{dR_1}{R_1}+\frac{d\Lambda_1}{\Lambda_1} \tag{S10}$$

Circumferential strain ε_θ is verified by the expression of strain in the cylindrical polar coordinates[2]:

$$\varepsilon_\theta=\frac{1}{r}\frac{\partial u_\theta}{\partial\theta}+\frac{u_r}{r} \tag{S11}$$

when

$$\varepsilon_\theta=\frac{dR_1}{R_1}+\frac{d\Lambda_1}{\Lambda_1}=\frac{1}{R_1}\frac{R_1 d\Lambda_1}{\Lambda_1}+\frac{dR_1}{R_1} \tag{S12}$$

In Eq. (S11), u_r, u_θ are radial and circumferential displacement, respectively.

Mechanics of OCS: rigid folding/unfolding

Analytical expressions of both the radial and axial balanced force are obtained with the method of minimum potential energy (equation (10)). Different load patterns and boundary conditions (Figs. S5a and S5b) determine the corresponding mechanical responses of folding/unfolding process (As a comparison, illustration of the line force and boundary conditions of inhomogeneous elastic deformation is also present in Fig. S5c). Both of the processes are implemented using the 9×5 OCS model. According to equation (10), external work induced by the isometric force is:

$$T=\int_0^{\chi_i} F_i\left(\chi_i\right)d\chi_i=\int_{\psi^{\text{init}}}^{\psi} F_i\left(\psi\right)\frac{d\chi_i}{d\psi}d\psi \tag{S13}$$

For the axial force, the positive direction is defined in the compressive/folding direction, so the displacement:

$$\chi_a=W^{\text{init}}-W=2ml\sin\alpha_1\left[\cos\left(\psi^{\text{init}}/2\right)-\cos\left(\psi/2\right)\right] \tag{S14}$$

For the radial force, the positive direction is also defined in the compressive direction, but note that a compressive load leads an unfolding process in this condition. Simple supported boundary condition is applied on the endpoints of R_1, so the radial displacement:

$$\chi_r=H^{\text{init}}-H \tag{S15}$$

where H is the rise of the shell structure (Fig. 3b), which can be described as:

$$H=R_2-R_1\cos\frac{n\lambda}{2} \tag{S16}$$

Note Equations (5) and (6):

$$R_i=l\frac{1}{\tan\alpha_2-\tan\alpha_1}\sqrt{\csc^2\left(\psi/2\right)+\tan^2\alpha_i},\left(i=1,2\right)$$

$$\lambda=\cos^{-1}\left[\frac{\tan^2\alpha_1\sin^2\left(\psi/2\right)-1}{\tan^2\alpha_1\sin^2\left(\psi/2\right)+1}\right]-\cos^{-1}\left[\frac{\tan^2\alpha_2\sin^2\left(\psi/2\right)-1}{\tan^2\alpha_2\sin^2\left(\psi/2\right)+1}\right]$$

Finally we obtain the non-dimensional radial force:

$$\bar{F}_r=\frac{F_r}{k}=\frac{2l\left[\left(2m-1\right)n\left(\psi-\psi^{\text{init}}\right)+m\left(n-1\right)G_1+mn\frac{\sin\alpha_1}{\sin\alpha_2}G_2\right]}{\partial\chi_r/\partial\psi} \tag{S17}$$

$$\overline{F}_a=\frac{F_a}{k}=\frac{-2}{\sin\alpha_1\sin\frac{\psi}{2}}\left[\left(2-\frac{1}{m}\right)n\left(\psi-\psi^{\text{init}}\right)+\left(n-1\right)G_1+n\frac{\sin\alpha_1}{\sin\alpha_2}G_2\right] \tag{S18}$$

Where

$$G_i=\frac{2\cos\alpha_i}{1-\sin^2\alpha_i\cos^2\frac{\psi^{\text{init}}}{2}}\left[\sin^{-1}\frac{\cos\alpha_i\cos\frac{\psi}{2}}{\sqrt{1-\sin^2\alpha_i\cos^2\frac{\psi}{2}}}-\sin^{-1}\frac{\cos\alpha_i\cos\frac{\psi^{\text{init}}}{2}}{\sqrt{1-\sin^2\alpha_i\cos^2\frac{\psi^{\text{init}}}{2}}}\right] \tag{S19}$$

Figure S5d shows the predicted $\overline{F_r}$ versus ψ of a 9×5 OCS with α_1=45°, $\alpha_2=60°$, and ψ^{init}=50°, respectively. The OCS can be completely unfolded with $\overline{F_r}$ increasing from 0 to about 90. While as it folds, $\overline{F_r}$ increases sharply at $\psi=\psi_c\approx 62.24°$. Further analysis of equation (17) shows that as $\psi\to\psi_c$, $\partial\chi_r/\partial\psi\to 0$,which means $F_r(\psi_c)\to\infty$. This phenomenon, therefore, is called "a limiting folding state ψ_c" in this paper. Note that ψ^{init} doesn't influence the value of ψ_c. Also included are the FEM simulated results (shown as blue dotted line in Fig. S5d). Excellent agreement between the analytical and FEM predictions is obtained. Figure S5e shows the variation of χ_r as ψ varies, which gives a geometric interpretation of the existence of ψ_c. Furthermore, by controlling parameters $m=9$ and $n=5$, we give a zero equipotential surface of $\partial\chi_r/\partial\psi$ as α_1 and α_2 change (α_1, α_2 should satisfy the closure conditions) to guide altering elastic properties and design paths of OCSs. As shown in Fig. S5f, every point on this surface indicates a limiting folding position and the corresponding (α_1, α_2). We can see from the phase paragraph that the phase diagram has 2 "branches", which indicates one single pair (α_1, α_2) may correspond to 2 different ψ_c if folding/unfolding starts from bottom/top, respectively.

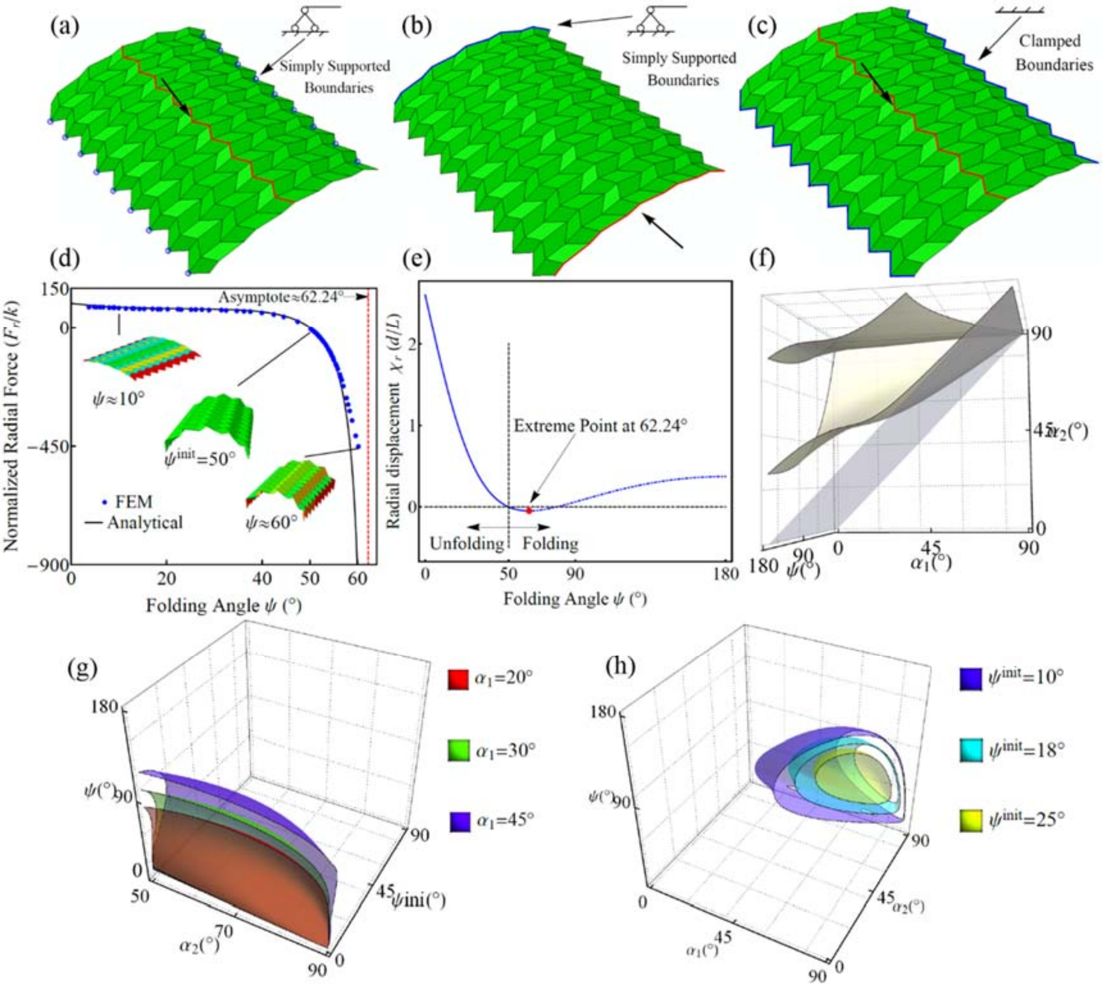

Figure S5 Mechanical responses of OCSs First 3 figures are different load patterns and boundary conditions, with red lines for force points, arrows for force directions and blue lines for boundaries, respectively. (a) Radial load and simply supported boundaries. (b) Axial load and simply supported boundaries. (c) Radial line force and clamped boundaries for deformation of elastic OCS. Figures in the second row are OCSs' responses under radial loads. (d) Analytical and FEM $\overline{F_r}-\psi$ curve of a 9×5 OCS under radial loads (α_1=45° , $\alpha_2=60°$, ψ^{init}=50°). 3 numerical folding states at $\psi\approx10°$, ψ^{init}=50° and $\psi\approx60°$ are illustrated inside, respectively. (e) $\chi_r-\psi$ curve of an OCS with $n=5$, $\alpha_1=45°$ and $\alpha_2=60°$. Monotonicity of χ_r determines existence of ψ_c . (f) The equipotential surface of $\partial\chi_r/\partial\psi$ when controlling $n=5$. Any straight line perpendicular to the bottom plane ($\alpha_1-\alpha_2$ plane) represents a folding/unfolding process. In the last row are additional information

mechanical responses of axial loads. (g) Equipotential surface expands as α_1 increases in $\alpha_2 - \psi^{\text{init}} - \psi$ space. (h) Equipotential surface shrinks as ψ^{init} increases in $\alpha_1 - \alpha_2 - \psi$ space.

FEM models of rigid folding and inhomogeneous deformation

For the rigid folding/unfolding process of an OCS, models consisting of rigid plates and linear elastic hinges are adopted. The properties of a creased sheet (Refer to Ref. 25 in the main text) is determined by the elastic constant k of the hinges, i.e., $k = cEh^3/12(1-v^2)$, where $c \approx 0.25$ is the torsion ratio, E =4 GPa is the Young's modulus, $v = 0.38$ is the Poisson's ratio and $h \approx 0.005m$ is the thickness of the sheet. In both analytical expressions and FEM simulations, side length of the OCS units L is set to be unit length for non-dimensionalization. It should be pointed out that, to model the plates in rigid folding/unfolding, a larger modulus of 100 GPa is employed in FEM simulations. Moreover, to connect large-modulus plates with linear elastic hinges and accurately quantify the hinges' elastic properties in FEM, programming language PYTHON is used as a secondary development tool for the simulation in ABAQUS. Some of the FEM modeling results are show in Fig. S6b. The associated PYTHON codes used to generate the models are available on request.

To realize different folding or deformation modes of the OCS, proper load patterns and boundary conditions need be carefully adopted, which are shown in Fig. S5. Shear deformable shell elements are used in the FEM. The equivalency is ensured by assuming that an OCS and its EHCS have the same amount of material, in addition to the same radius R_1 and central angle Λ_1, i.e.,

$$mn \cdot 4l_1 l_2 \sin\alpha_1 \cdot h_o = W \cdot R_1 \Lambda_1 \cdot h_e \tag{S20}$$

where h_o and h_e are the thicknesses of of the OCS and EHCS, respectively. Finally the thickness ratio of EHCS to OCS in FEM is:

$$\kappa = \frac{h_e}{h_o} = \frac{2\left(\tan\alpha_2 - \tan\alpha_1\right)}{\left(\theta_1 - \theta_2\right) \cdot \sqrt{\csc^2\left(\psi^{\text{init}}/2\right) + \tan^2\alpha_1} \cdot \cos\left(\psi^{\text{init}}/2\right)} \tag{S21}$$

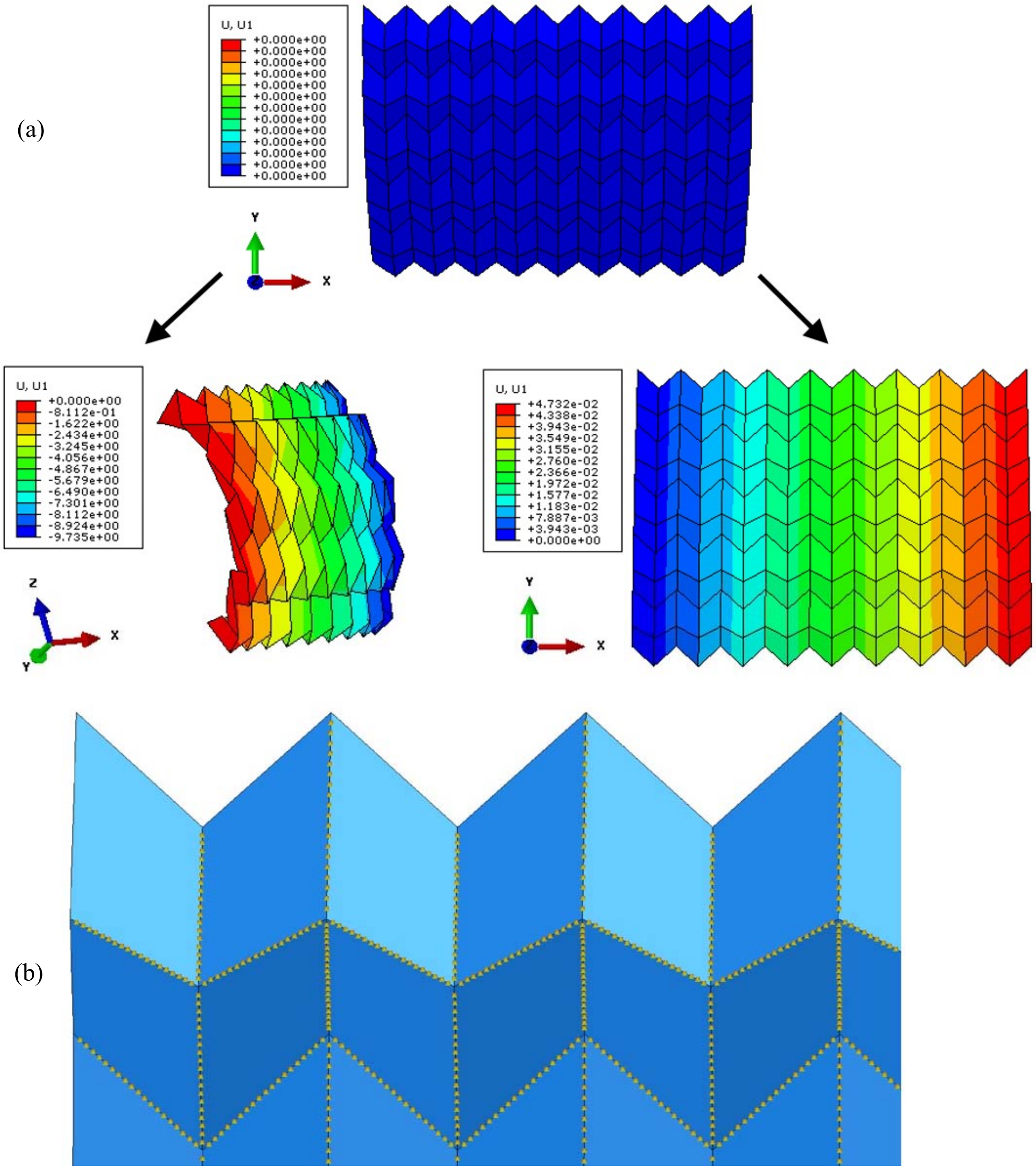

Figure S6 (a) Folding/unfolding process of a 9×5 rigid OCS in FEM, colors in which represent the displacement along x direction. (b) Modelling results of linear elastic hinges in FEM. Every little triangle represents a “discrete” hinge in the mesh.

References

1. Saito, K., Tsukahara, A. & Okabe, Y. New Deployable Structures Based on an Elastic Origami Model. *J. Mech. Des.* **137,** 021402 (2015).
2. Landau, L. D. & Lifshitz, E. M. *Theory of Elasticity*. (1986).
